# Expression Analysis of *Fyn* and *Bat3* Signal Transduction Molecules in Patients with Chronic Lymphocytic Leukemia

**DOI:** 10.31557/APJCP.2020.21.9.2615

**Published:** 2020-09

**Authors:** Fereshteh Hosseini-Valiki, Saeid Taghiloo, Golvash Tavakolian, Omolbanin Amjadi, Mohsen Tehrani, Akbar Hedayatizadeh-Omran, Reza Alizadeh-Navaei, Ehsan Zaboli, Ramin Shekarriz, Hossein Asgarian-Omran

**Affiliations:** 1 *Gastrointestinal Cancer Research Center, Non-Communicable Diseases Institute, Mazandaran University of Medical Sciences, Sari, Iran. *; 2 *Department of Immunology, School of Medicine, Mazandaran University of Medical Sciences, Sari, Iran. *; 3 *Student Research Committee, Mazandaran University of Medical Sciences, Sari, Iran. *; 4 *Department of Hematology and Oncology, Imam Khomeini Hospital, Mazandaran University of Medical Sciences, Sari, Iran. *

**Keywords:** Tim-3, Fyn, Bat3, exhausted T-cell, chronic lymphocytic leukemia

## Abstract

**Background:**

Chronic lymphocytic leukemia (CLL) is correlated with defects in T-cell function resulting imparity in antitumor immune responses. Tim-3 is a co-inhibitory immune checkpoint receptor expressed on exhausted T-cells during tumor progression. Fyn and Bat3 are two important adaptor molecules involved in inhibition and activation of Tim-3 downstream signaling, respectively. In this study, the expression of *Tim-3*, *Fyn*, and *Bat3 mRNA* was evaluated in CLL patients.

**Methods:**

Peripheral blood mononuclear cells (PBMCs) were isolated from 54 patients with CLL and 34 healthy controls. Total RNA was extracted from all samples and applied for cDNA synthesis. The relative expression of *Tim-3*, *Fyn*, and *Bat3 mRNA* was determined by TaqMan Real-Time PCR using GAPDH as an internal control.

**Results:**

*Tim-3 mRNA* expression was not significantly different between CLL patients and healthy controls. *Fyn mRNA* expression was significantly lower in CLL patients and conversely,* Bat3 mRNA* expression was higher in CLL patients compared to healthy controls. Interestingly, the *mRNA* expression of Fyn inhibitory adaptor molecule was remarkably associated with expression of *Tim-3* in CLL patients.

**Conclusion:**

We have highlighted for the first time the expression of *Fyn *and *Bat3* adaptor molecules in CLL patients. Our data demonstrated the strong correlation between the expression of Tim-3 and Fyn inhibitory molecules in CLL implying an important role for *Tim-3-Fyn* cooperation in induction of T-cell exhaustion.

## Introduction

Chronic lymphocytic leukemia (CLL) is correlated with main defects in T-cell function resulting imparity in antitumor immune responses and enhanced susceptibility to infections (Riches et al., 2013; Hanna et al., 2019). Recent surveys have shown that CLL patients have dysfunctional immune responses due to the T-cell exhaustion (Gorgun et al., 2009; Hofbauer et al., 2011; Riches et al., 2013). T-cell exhaustion is defined by a state of T-cell unresponsiveness which is generally caused by persistence of antigens in chronic conditions including infections and tumors (Mumprecht et al., 2009; Zhou et al., 2011; Parry et al., 2019). The exhaustion process is correlated with the defects of T-cell activities in terms of proliferation, cytotoxicity, cytokine secretion as well as up-regulation of several co-inhibitory receptors, such as programmed death-1 (PD-1), T-cell immunoglobulin and mucin-domain containing-3 (Tim-3), cytotoxic T lymphocyte associated protein-4 (CTLA-4), lymphocyte activation gene-3 (*LAG-3*), *CD244/2B4*, *CD160*, and T-cell immunoreceptor with Ig and ITIM domains (TIGIT) (Jin et al., 2010; Yi et al., 2010; Wherry, 2011). Among these co-inhibitory molecules, PD-1, and Tim-3 are two crucial immune checkpoint molecules which have been already established as the main T-cell exhaustion markers in several chronic pathological conditions including various types of solid and hematological malignancies (Sakuishi et al., 2010; Zhou et al., 2011; Llaó Cid et al., 2020). Besides PD-1, Tim-3 does not have a typical signaling motif in its cytoplasmic tail. There are five conserved tyrosine residues in the cytoplasmic tail of Tim-3 which Y256 and Y263 can be phosphorylated by either Src family kinases or interleukin-2-inducible T-cell kinase (ITK) (van de Weyer et al., 2006; Lee et al., 2011). Y256 and Y263 are implicated in the binding of phosphoinositide 3-kinases (PI3Ks) p85 subunit, Bat3 (HLA-B associated transcript 3), Fyn, and Lck (Lee et al., 2011; Rangachari et al., 2012). Bat3 binds to Y256 and Y263 of Tim-3 in the lack of its signaling with corresponding ligands and prevents SH2 domain-binding sites in the Tim-3 tail. In this situation, Bat3 employs the active form of Lck promoting an intracellular molecular complex with Tim-3 that effectively stimulates T-cell signaling (Rangachari et al., 2012). Galectin-9 binding to Tim-3, as the major ligand of this receptor, results in phosphorylation of Y256 and Y263 and releasing of Bat3 from the Tim-3 tail, thus stimulating Tim-3 mediated suppression of T-cells by promoting binding of Src kinases and modulation of TCR signaling (Rangachari et al., 2012; Huang et al., 2015). Notably, like Bat3, Fyn adaptor molecule binds to the same region on the Tim-3 tail and is involved in the initiation of T-cell exhaustion or anergy (Davidson et al., 2007). So, it is possible that a change between Tim-3-Bat3 to Tim-3-Fyn can cause the switch of Tim-3 function from stimulation to suppression of TCR signaling. So, a crucial determinant of the Tim-3 activity may be aroused from the balance between Fyn and Bat3 bounded to the Tim-3 intracellular tail (Anderson et al., 2016).

Accordingly, the expression of *Tim-3* has been studied in a variety of cancers as well as in patients with CLL, but the expression of *Fyn* and *Bat3*, as the main down-stream adaptor molecules of Tim-3, has not been investigated in this leukemia. The aim of this study is to evaluate the expression of *Tim-3* adapter molecules, Fyn and Bat3, in CLL patients which could help identifying immune regulation mechanisms in this malignancy and introducing novel immunotherapy targets to enhance the response of T-cells in these patients.

## Materials and Methods

*Patients and healthy controls*

Peripheral blood was obtained from 54 patients with CLL, who had not received any chemotherapy regimen, attending the Hematology and Oncology Clinic of Imam Khomeini Hospital, affiliated to Mazandaran University of Medical Sciences, and 34 age- and sex-matched healthy controls. Patients were clinically classified based on the Rai staging system; 41 patients in early stages (stages 0 and I) and 13 patients in advanced stages (stages II, III, and IV). Written informed consents were taken from all participants and the study was approved by the Ethical Committee of Mazandaran University of Medical Sciences. CLL diagnosis was done based on the clinical examination, peripheral blood cell count, cell morphology, and immunophenotyping analysis according to the criteria outlined by WHO (Pardoll, 2012). Major clinical and hematological characteristics of patients are summarized in Table 1.

*RNA isolation and cDNA synthesis*

Peripheral blood mononuclear cells (PBMCs) were isolated from all participants via Ficoll-Histopaque density gradient centrifugation. Total RNA was extracted from PBMCs using RNA extraction kit (DenaZist Asia, Mashhad, Iran) based on the manufacturer’s protocol. The quality of extracted RNA was confirmed by electrophoresis and nano-spectrophotometer. Complementary DNA (cDNA) was reverse-transcribed from 1 microgram of total RNA using Thermo Scientific RevertAid first strand cDNA synthesis kit (Thermo Scientific, Massachusetts, USA) in a 20μl reaction mixture containing 4μl of 5x reaction buffer, 1μl random hexamer primer, 2 μl dNTP 10mM, 200 unit RevertAid M-MuLV reverse transcriptase enzyme, 1μl RNase inhibitor, and appropriate RNase/DNase free water. The mixture was then incubated at 25°C for 5 min, 42°C for 1 hour and 70°C for 5 min.

*Semi-quantitative Real-Time PCR*

Real-Time PCR was performed by iCycler iQ5 Real-Time PCR system (Bio-Rad, California, USA) to analyze the gene expression using TaqMan-based primers and probes for Tim-3, *Fyn*, *Bat3*, and the internal control gene *GAPDH*. The sequence for primers and probes are given in Table 2. Each gene was amplified using an RT-PCR reaction mix prepared with 2 µl TaqMan primers/probe mix, 10 μl of TaqMan master mix, 2 μl of cDNA template, and ultrapure DNase/RNase-free distilled water. PCR reactions were amplified at 95°C for initial denaturation followed by 40 cycles at 94°C for 30 seconds, 57°C for 30 seconds, and extension at 72°C for 30 seconds. Finally, the relative expression level of Tim-3, Fyn, and Bat3 was calculated with 2^-ΔCt^ value using *GAPDH* as a housekeeping gene.

*Statistical analysis*

All statistical analyses and graphs were performed by GraphPad Prism 6 software. Kolmogorov-Smirnov test was done to determine the normality distribution of the data. Mann-Whitney U test was applied to compare the mean differences between two groups. Finally, for correlation analysis, Spearman’s rank correlation test was used. Data are expressed as median ± interquartile range (IQR) and p-values less than 0.05 were considered significant. 

## Results

*Expression levels of Tim-3, Fyn, and Bat3 in CLL patients and healthy controls*

**Figure 1:**
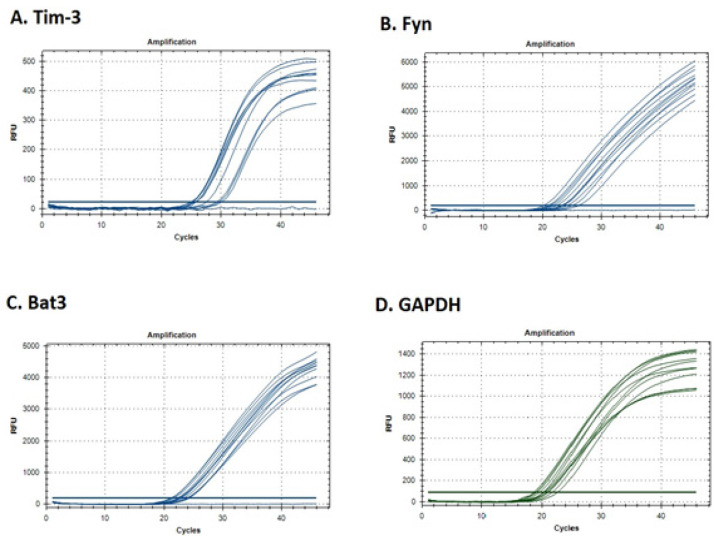
Amplification Curve Analysis of *Tim-3* (A), *Fyn *(B), *Bat3* (C), and GAPDH (D) mRNA Expression Obtained from Real-Time PCR Assay

**Figure 2:**
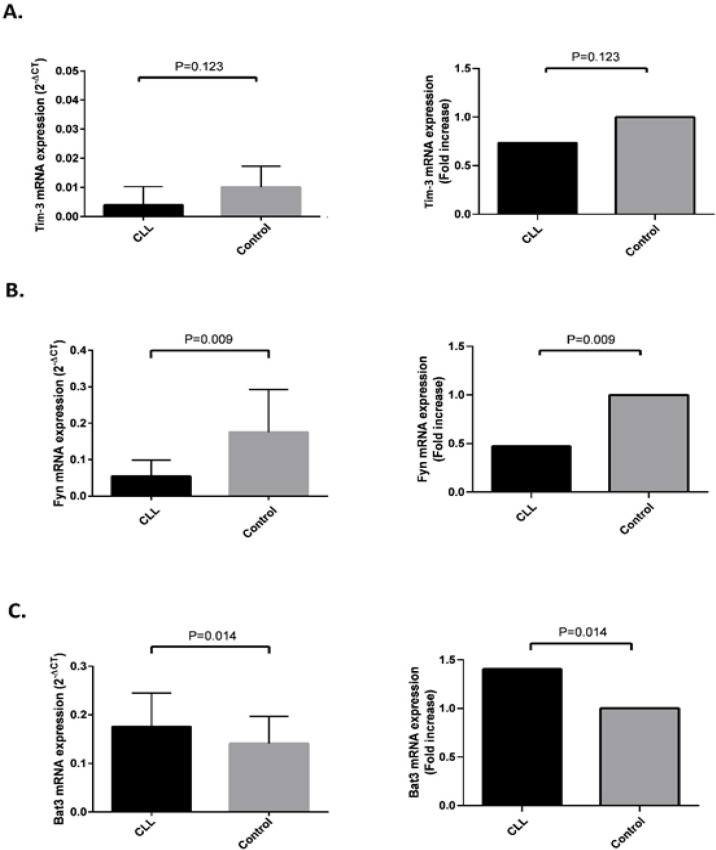
Expression Profile of Tim-3 (A), Fyn (B), and Bat3 (C) mRNA in CLL Patients and Normal Individuals. Total RNA was extracted from peripheral blood and single-strand cDNA was synthesized. TaqMan Real-Time PCR was performed with specific primers and probes to measure the mRNA expression of Tim-3, Fyn, Bat3, and GAPDH. All experiments were performed in duplicate and the results are expressed as the median ± IQR of 2^-ΔCt^ and fold increase in patients compared to controls after normalization with GAPDH as an internal control. P-values < 0.05 were considered significant

To examine the* mRNA* expression profile of Tim-3, Fyn and Bat3, PBMCs were isolated from CLL patients and healthy controls. The relative *mRNA* expression of the mentioned genes was investigated using a Real-Time PCR method using GAPDH as an internal control. Amplification curve analysis results obtained for Tim-3, Fyn, Bat3, and GAPDH are illustrated in Figure 1. Our findings showed that *Tim-3* expression was not significantly different in CLL patients in comparison with healthy controls (p = 0.123, Figure 2A), but *Fyn *expression was significantly decreased in CLL patients (p = 0.009, Figure 2B). Moreover, the up-regulation of Bat3 molecule was seen in CLL patients in comparison with healthy controls (p = 0.014, Figure 2C). We further analyzed the correlation of *Tim-3*, *Fyn*, and *Bat3* expression with clinical stages of CLL patients. However, the expression of these molecules was not significantly different in patients at early and advanced clinical stages of the disease.

*Fyn expression was positively correlated with Tim-3 expression in CLL patients*

**Figure 3:**
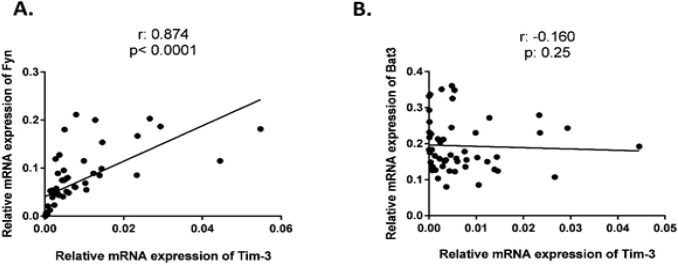
Correlation Analysis of Fyn and Bat3 mRNA Expression with the mRNA Expression of Tim-3 in CLL Patients. A. Fyn mRNA expression was strongly associated with the mRNA expression of Tim-3 in CLL patients (r= 0.874, p<0.0001). B. Bat3 mRNA expression was not significantly correlated with the mRNA expression of Tim-3 in CLL patients (r= -0.160, p=0.25)

Since Fyn and Bat3 bind to the same domain of the Tim-3 cytoplasmic region resulting inhibition and activation of Tim-3 signaling, respectively, so the *mRNA* expression results were analyzed to find any correlations between *Fyn* and *Bat3 mRNA* expression with the expression of *Tim-3* in CLL patients. Interestingly, *Fyn* *mRNA* expression was strongly associated with the *mRNA *expression of Tim-3 in CLL patients confirming remarkable recruitment of Fyn by Tim-3 to inhibit the response of T-cells in these patients (Figure 3A, r= 0.874, p<0.0001). However, *Bat3 mRNA* expression was not correlated with the *mRNA* expression of Tim-3 in CLL patients (Figure 3B, r=-0.160, p=0.25). 

**Table 1:** Major Clinical and Laboratory Characteristics of CLL Patients and Healthy Controls

Characteristics	CLL Patients (n=54)	Normal Controls (n=34)	*p*-value
Number of subjects	54	34	
Gender	Male (31)	Male (23)	> 0.05
	Female (24)	Female (12)	
WBC × 10^3^ /mm^3^			
Mean ± SEM	43.2 ± 4.01	7.2 – 0.33	<0.0001
Range	4.2 – 170.1	3.8 – 11.4	
Lym (%)			
Mean ± SEM	79.5 ± 1.64	38.2 ± 1.01	<0.0001
Range	55.9 – 95	27.6 – 53.20	
PLT×10^3^/ mm^3^			
Mean ± SEM	163.9 ± 8.92	204.7 ± 13.44	0.008
Range	16 – 365	120 – 290	
Hb (g/dl)			
Mean ± SEM	12.29 ± 0.28	13.35 ± 0.26	0.02
Range	6 – 16.10	10.10 – 16.10	

CLL, chronic lymphocytic leukemia; WBC, white blood cell count; Lym, lymphocytes present in peripheral blood; Hb, hemoglobin; PLT, platelet count; SEM, standard error of mean; p-values < 0.05 were considered significant.

**Table 2:** Sequences of Primers and Probes Used in Real-Time PCR mRNA Analysis

Target	Sequences	Conjugate dye
Tim-3		FAM
Forward	5'- CCAGCAGAGACACAGACACT -3'	
Reverse	5'- TTGCTCCAGAGTCCCGTAAG -3'	
Probe	5'- GGCCAATGAGTTACGGGACT-3'	
Bat3		FAM
Forward	5'- TTCTTTGGGGCCTTGCTTTC -3'	
Reverse	5'- ACTCTCCCGCACATACTCTT -3'	
Probe	5'- CAGCTGCGATCCTTCTTCCA-3'	
Fyn		FAM
Forward	5'- TGTGGCTCCAGTTGACTCTA-3'	
Reverse	5'- CCACCATTGTCAAGTTTGCG-3'	
Probe	5'- CCGAAAAGATGCTGAGCGAC-3'	
GAPDH		HEX
Forward	5'- GGGTGTGAACCATGAGAAGT -3'	
Reverse	5'- GCAGGGATGATGTTCTGGAG -3'	
Probe	5'- TCGTGGAAGGACTCATGACC-3'	

## Discussion

Co-inhibitory receptors expressed on exhausted T-cells have been appeared as crucial goals for tumor immunotherapy (Pardoll, 2012; Nirschl and Drake, 2013; Llaó Cid et al., 2020). In the tumor microenvironment, exhausted T-cells are determined by impaired functional activities like cytokines secretion and up-regulation of co-inhibitory molecules, including CTLA-4, PD-1, Tim-3, and LAG-3 (Wherry, 2011). Based on the association of *Tim-3* expression with exhaustion process of T-cells, it has been introduced as a main target for the development of antitumor immunotherapy (Blackburn et al., 2009; Ngiow et al., 2011; Zhou et al., 2011). However, signal transduction molecules and signaling pathways in downstream of Tim-3 are still unclear. Therefore, to further explore the signal transduction molecules in Tim-3 co-inhibitory pathways in patients with CLL, we examined the expression of *Fyn* and* Bat3* as the potential signal transduction molecules in downstream of Tim-3 signaling. Interestingly, the expression of inhibitory* Fyn* adaptor molecule was significantly associated with *Tim-3* expression in patients with CLL. To our knowledge, this is the first data which indicates the expression of *Fyn* and *Bat3* in *CLL* patients.

It has been demonstrated that Tim-3 is over-expressed on T-cells of several solid and hematological malignancies (Sakuishi et al., 2010; Riches et al., 2013; Hadadi et al., 2019). In agreement to this finding, our recent studies displayed more expression of* Tim-3* and *PD-1* on both CD4+ and CD8+ T cells from patients with CLL (Allahmoradi et al., 2017; Taghiloo et al., 2017). But in contrast with these findings, in this study, we did not find any differences between the *Tim-3 mRNA* expression in CLL patients and healthy controls. The main reason for this discrepancy is because of different samples used for *Tim-3* expression analysis. Here, Tim-3 mRNA is measured in PBMC of CLL patients, but in our previous studies, a three-color flow cytometry method was applied to determine the Tim-3 protein expression on CD4+ and CD8+ T-cells of CLL patients. Altogether, if we were able to measure the *Tim-3 mRNA* expression in isolated CD4+ or CD8+ T-cells, the obtained results from *mRNA* expression analysis might be the same as the protein expression data. 

In order to clarify how Tim-3 can modify T-cells function, various studies examined the signaling pathways associated with Tim-3 receptor (Tomkowicz et al., 2015). A preserved tyrosine residue, Y263, located in the Tim-3 cytoplasmic tail, is recognized to be phosphorylated by inducible T-cell kinase (ITK) following ligation with galectin-9 in HEK-293T cells (van de Weyer et al., 2006). There has been disagreement between studies as to whether Tim-3 activates or suppresses T-cells activity. Furthermore, the molecular mechanisms controlling the role of Tim-3 signaling remain poorly understood. Fyn adaptor molecule is a member of the Src family of kinases that binds to the same region on the Tim-3 tail as Bat3. Fyn tyrosine kinase has a pivotal role in different biological processes, such as cell growth, differentiation, and T-cell signaling, being also implicated in the pathogenesis of solid tumor and hematologic malignancies including cancer cell invasion, metastasis, proliferation, survival and angiogenesis (Palacios and Weiss, 2004; Yadav and Denning, 2011; Singh et al., 2012; Laurenzana et al., 2016)*. Fyn* expression is up-regulated in several solid tumors, including glioblastoma, head and neck squamous cell carcinoma, melanoma, pancreatic, prostate cancer, and squamous cell carcinoma (Ayli et al., 2008; Ban et al., 2008; Zhao et al., 2009; Yadav and Denning, 2011). Fyn involvement has also been shown in hematological malignancies, including chronic myeloid leukemia (CML), acute myeloid leukemia (AML), acute lymphoid leukemia (ALL), multiple myeloma, and T cell lymphomas which is associated with poor prognosis (Palomero et al., 2014; Chougule et al., 2016; Laurenzana et al., 2016). Galectin-9, as the major ligand of Tim-3, mediates the release of Bat3 from Tim-3 through phosphorylation of Y256 and Y263 in the Tim-3 cytoplasmic tail (Rangachari et al., 2011). It was proved that Bat3 binding to the intracellular domain of the Tim-3 tail, recruit the active form of Lck leads to T-cell activation and suppressing exhaustion (Rangachari et al., 2012). Intriguingly, an exhaustion phenotype is caused by down-regulation of Bat3 in Th1 cells. Reduced expression of *Bat3* in T-cells associated with decreasing in their effector functions, like proliferation and cytokines production as well as enhancing in expression of some co-inhibitory receptors involved in exhaustion process including *Tim-3, LAG-3, *and *IL-10* (Rangachari et al., 2012; Ji et al., 2018). These results are in contrast with our findings indicating the higher expression of* Bat3* and lower expression of *Fyn* in CLL patients compared to controls. This controversy is highly related to the sample type applied for *mRNA *expression analysis. In the current study*, mRNA* expression levels of *Tim-3, Fyn*, and *Bat3 *were measured in PBMC of CLL patients and normal controls which are completely different in terms of T-cells percentage. While leukemic B-cells are the most frequent cells in PBMC obtained from CLL patients, samples from normal controls are predominantly consisted of CD4+ and CD8+ T-cells. Using isolated CD4+ or CD8+ T-cells for *mRNA* expression analysis could solve this problem and leads to get more different data. But, to isolate appropriate number of CD8+ T-cells for RNA extraction and cDNA synthesis, we needed 15-20 ml whole blood from CLL patients which was not ethically possible. Besides this limitation, our data demonstrated that the expression of *Fyn* inhibitory adaptor molecule is highly associated with Tim-3 expression in CLL patients confirming the more recruitment of Fyn by Tim-3 in CLL to induce the exhaustion process of T-cells for evasion of leukemic cells from host immune responses. According to the results obtained from several studies, Fyn binding to intracytoplasmic domain of Tim-3 enhances the exhaustion process and inhibitory function of T-cells. Moreover, higher expression of *Bat3* in CLL patients may be related to the some unknown roles of this adaptor molecule in CLL pathogenesis which needs to be clarified in future studies. Nonetheless, further works are needed to better understand the roles of the Bat3/Tim-3 and Fyn/Tim-3 interactions in the process of T-cell exhaustion in CLL patients.

In conclusion, we have highlighted for the first time the expression of *Bat3* and *Fyn* adaptor molecules in CLL patients. However, our data demonstrated the strong correlation between the expression of *Fyn* inhibitory adaptor molecule and Tim-3 in CLL. This data imply an important role for Fyn-Tim-3 axis in induction of T-cell exhaustion in CLL patients. Accordingly, considering of the Fyn/Tim-3 and Bat3/Tim-3 axes could be a promising molecular target for therapeutic interventions in hematological malignancies like CLL.
